# Highly diverse cuticular hydrocarbon profiles but no evidence for aggression towards non‐kin in the ambrosia beetle *Xyleborinus saxesenii*

**DOI:** 10.1002/ece3.11274

**Published:** 2024-04-23

**Authors:** Antoine Melet, Viesturs Leibold, Thomas Schmitt, Peter H. W. Biedermann

**Affiliations:** ^1^ Chair of Forest Entomology and Protection, Faculty of Environment and Natural Resources Albert‐Ludwigs‐Universität Freiburg Germany; ^2^ Department of Animal Ecology and Tropical Biology, Biocentre University of Würzburg Würzburg Germany

**Keywords:** chemical communication, cooperative breeding, cuticular hydrocarbons, nestmate recognition

## Abstract

Animal societies use nestmate recognition to protect against social cheaters and parasites. In most social insect societies, individuals recognize and exclude any non‐nestmates and the roles of cuticular hydrocarbons as recognition cues are well documented. Some ambrosia beetles live in cooperatively breeding societies with farmed fungus cultures that are challenging to establish, but of very high value once established. Hence, social cheaters that sneak into a nest without paying the costs of nest foundation may be selected. Therefore, nestmate recognition is also expected to exist in ambrosia beetles, but so far nobody has investigated this behavior and its underlying mechanisms. Here we studied the ability for nestmate recognition in the cooperatively breeding ambrosia beetle *Xyleborinus saxesenii*, combining behavioural observations and cuticular hydrocarbon analyses. Laboratory nests of *X. saxesenii* were exposed to foreign adult females from the same population, another population and another species. Survival as well as the behaviours of the foreign female were observed. The behaviours of the receiving individuals were also observed. We expected that increasing genetic distance would cause increasing distance in chemical profiles and increasing levels of behavioural exclusion and possibly mortality. Chemical profiles differed between populations and appeared as variable as in other highly social insects. However, we found only very little evidence for the behavioural exclusion of foreign individuals. Interpopulation donors left nests at a higher rate than control donors, but neither their behaviours nor the behaviours of receiver individuals within the nest showed any response to the foreign individual in either of the treatments. These results suggest that cuticular hydrocarbon profiles might be used for communication and nestmate recognition, but that behavioural exclusion of non‐nestmates is either absent in *X. saxesenii* or that agonistic encounters are so rare or subtle that they could not be detected by our method. Additional studies are needed to investigate this further.

## Graphical abstract

We confirmed that different populations of *Xyleborinus saxesenii* have different chemical profiles. However, we found no evidence for different behavioural responses in relation to these chemical differences, which is surprising considering the typical, highly aggressive response displayed by other social insects in similar situations.

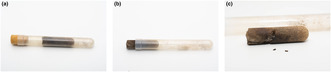

## 1 INTRODUCTION

Animal societies are vulnerable to cheaters and social parasites. To prevent invasion, most of them have group‐recognition mechanisms in order to exclude any individual that is not part of the nest community (Hölldobler & Wilson, 1990). To discriminate between nestmates and non‐nestmates, social animals perceive and respond to an array of sensory cues. The best studied systems are the visual and acoustic cues used by cooperatively breeding birds and the chemical cues used by (eu)social insects (Rossi & Derégnaucourt, 2020). In social insects, unsaturated and methyl‐branched hydrocarbons play a major role in communication (Drijfhout et al., 2013). However, there is also evidence for the use of chemical cues by birds (Krause et al., 2012) and visual cues by wasps (Cini, Cappa, et al., 2019; Tibbetts, 2002).

Ambrosia beetles (Curculionidae: Platypodinea and Scolytinae) live within dark, closed nests, dug into dying or dead wood, where they farm fungi on the nest walls (Biedermann & Vega, 2020; Hulcr & Stelinski, 2017). The scolytine tribe Xyleborini is the most species‐rich. It includes species that practice inbreeding and live in communal societies where adults delay dispersal and feed on their cultivated fungal crops with the developing larvae (Kirkendall et al., 2015). In very few species, including our model species, the fruit‐tree pinhole borer, *Xyleborinus saxesenii* Ratzeburg (Scolytinae; Xyleborini), it has been shown that the adult daughters of a nest‐founding female not only delay dispersal from their natal nest but also actively engage in cooperative tasks like brood care, nest hygiene and protection as well as fungus‐farming (Biedermann & Taborsky, 2011; Nuotclà et al., 2019). Some of these daughters also reproduce alongside their mother in the natal nest (Biedermann, Peer, & Taborsky, 2011) and there is evidence that these females engage more strongly in cooperative tasks than the non‐breeding helping daughters (in the related species *Xyleborus affinis* Eichhoff) (Biedermann, 2020). When fungal food becomes scarce, all daughters disperse and find their own nests (Biedermann, Klepzig, & Taborsky, 2011; Biedermann, Peer, & Taborsky, 2011; Biedermann & Taborsky, 2011; Moser & Taborsky, 2011; Peer & Taborsky, 2007). While unique for beetles, this is a typical cooperative breeding system (Kirkendall et al., 2015).

The foundation of a new nest is the most critical time in the life cycle of a female ambrosia beetle. Nest‐founding females have to find a suitable tree host, bore a labour‐intensive tunnel in the xylem, inoculate tunnel walls with the spores of their fungal mutualists and take care of the fungal cultures. Even though nest‐founding success seems to vary among species, both field and laboratory data for *X. saxesenii* showed that, on average, only about 20% of the nest‐founding females successfully start their food‐fungus cultures and produce offspring (Biedermann et al., 2009; Peer & Taborsky, 2007). Once the fungus grows and the first batch of eggs is produced, it is almost guaranteed that the offspring will develop into adults (Biedermann et al., 2009). Given the high payoff of sneaking into an already established nest without paying the cost of a risky nest foundation, it is possible that social cheaters exist who may try to enter foreign nests.

All species in the tribe of *Xyleborini* ambrosia beetles were thought to be obligate inbreeders because mating has been solely observed between brothers and sisters in their natal nests (Biedermann, 2010; Jordal et al., 2001). However, genetic studies have recently shown that outbreeding must exist and is more common than expected because the estimated inbreeding indices in *Xylosandrus crassiusculus* are lower than expected for a completely inbred species (Storer et al., 2017). This suggests that individuals can enter foreign nests or that neighbouring nests merge more frequently than currently envisioned. In any case, foreign individuals may potentially exploit local resources, increase conflict and thus reduce the fitness of native individuals (Bourke, 2011; Ratnieks et al., 2006; Schultner et al., 2014). Alternatively, non‐nestmate conspecifics may be allowed to enter the nest and mate to increase genetic diversity because it can make insect societies more resistant against pathogens (Sherman et al., 1988) and parasites (Shykoff & Schmid‐Hempel, 1991). Indeed, *Ips typographus*, another outbreeding scolytine beetle, was found to mate preferentially with non‐relatives (Dacquin et al., 2023). Cases of social parasitism are known from other social insects like bees (Beekman & Oldroyd, 2008; Härtel et al., 2006; Nanork et al., 2006; Wenseleers et al., 2011), bumblebees (Birmingham et al., 2004; Lopez‐Vaamonde et al., 2004), wasps (Cervo, 2006; Cini, Sumner, et al., 2019; Oliveira et al., 2016), ants (Dobata et al., 2009) and gall‐forming thrips. In the latter, for example, *Koptothrips* species invade galls made by their *Kladothrips*, *Oncothrips* or *Onychothrips* hosts (Crespi, 1992; Mound, 1971). Despite having a similar fortress‐defender social system as the thrips, such social parasitism is not known to exist in ambrosia beetles. Nestmate recognition protects against such exploitation and is ubiquitous in insect societies (Crozier & Pamilo, 1996; Van Zweden & d'Ettorre, 2010). Other social insect species use behavioural, chemical, architectural and morphological defences (Grüter et al., 2018). Ambrosia beetles exhibit morphological defences because the single entrance tunnels of nests are typically well protected by females that physically block the entrance with their bodies against any intruder (Biedermann & Taborsky, 2011; Nuotclà et al., 2014). However, whether individuals also react to already entered foreign individuals is unknown.

In other social insects, the most commonly studied defence against non‐nestmates is aggressiveness, which may even be lethal (Breed, 2014; Gamboa, 2004; Sturgis & Gordon, 2012; Tsutsui, 2004). Members of a nest discriminate nestmates from non‐nestmates and either keep the non‐nestmates from entering the nest or immediately expel already entered individuals. Behavioural reactions involve a nestmate label, the perception of this label, the comparison of this label to an internal template and a suit of typically aggressive behaviours specifically targeting non‐nestmates (Cappa et al., 2020; Gamboa, 2004; Signorotti et al., 2014). In ambrosia beetles, cannibalism towards larvae or adult nestmates is often observed (Biedermann & Taborsky, 2011), and it is possible that aggressive behaviours are specifically addressed towards non‐nestmates, as observed in many other social insects (Beye et al., 1998; Blight et al., 2012; Drescher et al., 2010; Frizzi et al., 2014; Holzer et al., 2006; Sturgis & Gordon, 2012; Vásquez & Silverman, 2008). Non‐aggressive behavioural reactions (such as reduced grooming and feeding) towards non‐nestmates may also be elicited, but these are generally poorly studied in social insects (Björkman‐Chiswell et al., 2008).

Cuticular hydrocarbon (CHC) profiles may carry information about individual castes, sex, reproductive status and dominance position (Bagnères & Blomquist, 2010; Beani et al., 2019) and are the main chemical cues used by social insects to label nestmates (Akino et al., 2004; Howard & Blomquist, 1982, 2005; Lahav et al., 1999; Singer, 1998; Sturgis & Gordon, 2012; Van Zweden & d'Ettorre, 2010). An individual compares the profile perceived during antennation to a nest‐specific template to discriminate between nestmates and non‐nestmates and larger differences between the template and the profile elicit stronger behavioural responses (Blight et al., 2012; Jutsum et al., 1979; Vásquez & Silverman, 2008). In a population, differences between colonies can already elicit aggressive responses. However, individuals originating from the same population are expected to have relatively similar CHC profiles that provoke less or a different nature of behavioural responses, while individuals from different populations are expected to have more distant CHC profiles and may elicit more intense behavioural responses. Individuals from a different species should be even more chemically distant and elicit the strongest behavioural response.

This research examines, for the first time, whether nestmate recognition is present in ambrosia beetles and if it is based on CHC profile differences. We analysed the CHC profiles of two *X. saxesenii* populations under the assumption that differences in their profiles may provide cues for nestmate recognition. We also studied the responses of *X. saxesenii* towards conspecifics from the same population, conspecifics from a different population and individuals from a different ambrosia beetle species (i.e., *Xylosandrus germanus*). Behaviours and the survival of introduced adult females were compared between these treatments.

## 2 MATERIALS AND METHODS

### 2.1 Collection of beetles

All beetles used in our experiments originated from three laboratory stock lines that were initiated with beetles caught in the wild in June and July of 2019 at two sites located 300 km apart. The first *X. saxesenii* line was initiated by beetles actively dispersing, caught in the Steinbachtal forest (SBT) in Würzburg, Germany (GPS coordinates: 49.771592, 9.921924) using ethanol‐baited traps. This forest is dominated by beech trees, other deciduous species and a few coniferous trees. A second *X. saxesenii* line was initiated with fully sclerotised beetles collected in their nests in the Bavarian Forest (BW) National Park (Bayerischer Wald, BW) close to Neuschönau, Germany (GPS coordinates: 48.897134, 13.499431) by opening nests found within dead beech (*Fagus sylvatica*) wood. This forest is dominated by fir trees with beech (Bayerische Landesanstalt für Wald und Forstwirtschaft, 2002). Observation of the laboratory stock lines confirms that beetles are already able to find their own nest before they disperse and that beetles actively dispersing do not perform differently than relatively old, sclerotised beetles collected in their nests. For the third breeding line, we used adult females of the species *X. germanus* caught in SBT.

### 2.2 Artificial rearing

All female beetles used for laboratory rearing were first washed in 70% ethanol for a few seconds to eliminate environmental contamination of bacteria and fungi from the body surface of the beetles and then rinsed in distilled water to remove the toxic ethanol. Since *X. saxesenii* carry the spores of their primary fungal mutualists in the mycetangia and gut (Francke‐Grosmann, 1975), they are not harmed by the sterilisation of the outer surface (Biedermann et al., 2009). We did not record the number of colonies successfully established for each population at the time. Afterwards, the beetles were dried on sterile tissue paper and immediately, individually placed on artificial medium in plastic tubes that were then closed with foam plugs. The rearing medium consists of sawdust, agar and nutrients (see “standard medium” in Biedermann et al., 2009). This procedure was repeated for each laboratory generation. Dispersing adult females were collected from the surface of the medium in the rearing tubes, sterilised and then individually placed on fresh artificial medium in new tubes. All nests were kept at a constant temperature of 25°C, 70% humidity and in constant darkness.

This artificial rearing protocol is highly successful, and as a result, we have beetles in high numbers in the laboratory. Part of the laboratory population was used for this experiment. We did not record the exact proportion of nests out of our lab populations that we used for this experiment.

### 2.3 CHC analysis

For CHC analyses exclusively laboratory‐reared beetles were used. One hundred dispersing adult female *X. saxesenii* from the SBT population and 100 adult females from the BW population (each from the 2nd and 3rd laboratory generations of the respective lines) were collected and prepared for gas chromatography/mass spectroscopy (GC–MS) analysis. Each beetle was killed by freezing and stored at −20°C before extraction. As adult females are small (less than 2 mm long), 20 individuals were pooled for every CHC extraction to get sufficient amounts of hydrocarbons. This means that each population was replicated five times. For each replicate, 20 frozen females from either the SBT or BW population were submerged in hexane in 4 mL glass vials for 15 min. The hexane was completely evaporated under a gentle flow of CO_2_. The resulting dry fraction was then resuspended in 100 μL of hexane. We could not extract the CHCs of the individuals after their introduction into foreign nests because the paint used for marking would have contaminated the chemical profiles. Furthermore, due to the limited number of beetles, we could not extract the CHCs of *X. germanus*.

We used a 7890A GC System coupled to a 5975 Mass Selective Detector and a 7693 Autosampler (Agilent Technologies, Waldheim, Germany) for the gas chromatography. The GC was equipped with a J&W DB‐5 fused silica capillary column (30 m × 0.25 mm ID; df = 0.25 μm; J&W, Folsom, CA, USA). The temperature was programmed to increase from 60 to 300°C in increments of 5°C/min and kept at 300°C for 10 min. Helium was used as the carrier gas with a constant flow rate of 1 mL/min. Injection was carried out at 300°C in splitless mode for 1 min. The electron impact mass spectra were recorded at 70 eV. We used ChemStation software (Agilent Technologies, Böblingen, Germany) for data acquisition and processing. Hydrocarbons were identified using diagnostic ions and retention indices (Carlson et al., 1998).

Only non‐polar, non‐volatile compounds were included in the analysis of the cuticular profiles. Polar compounds do not vary between colonies of *Polistes dominulus* wasps and are not used for nestmate recognition (Bruschini et al., 2011). Generally, the role of polar compounds in nestmate recognition is unclear. To investigate the role of polar compounds was beyond the scope of this study, so we did not include them in our analysis. Additionally, the only polar compounds we found were volatiles that cannot function as nestmate recognition cues since they disappear over time. Volatile compounds were not recorded in our dataset. Hydrocarbons that accounted for less than 0.01% of the total peak area were excluded to avoid the effect of small concentration differences on the analyses.

### 2.4 Individual marking and introduction in a nest

**FIGURE 1:**
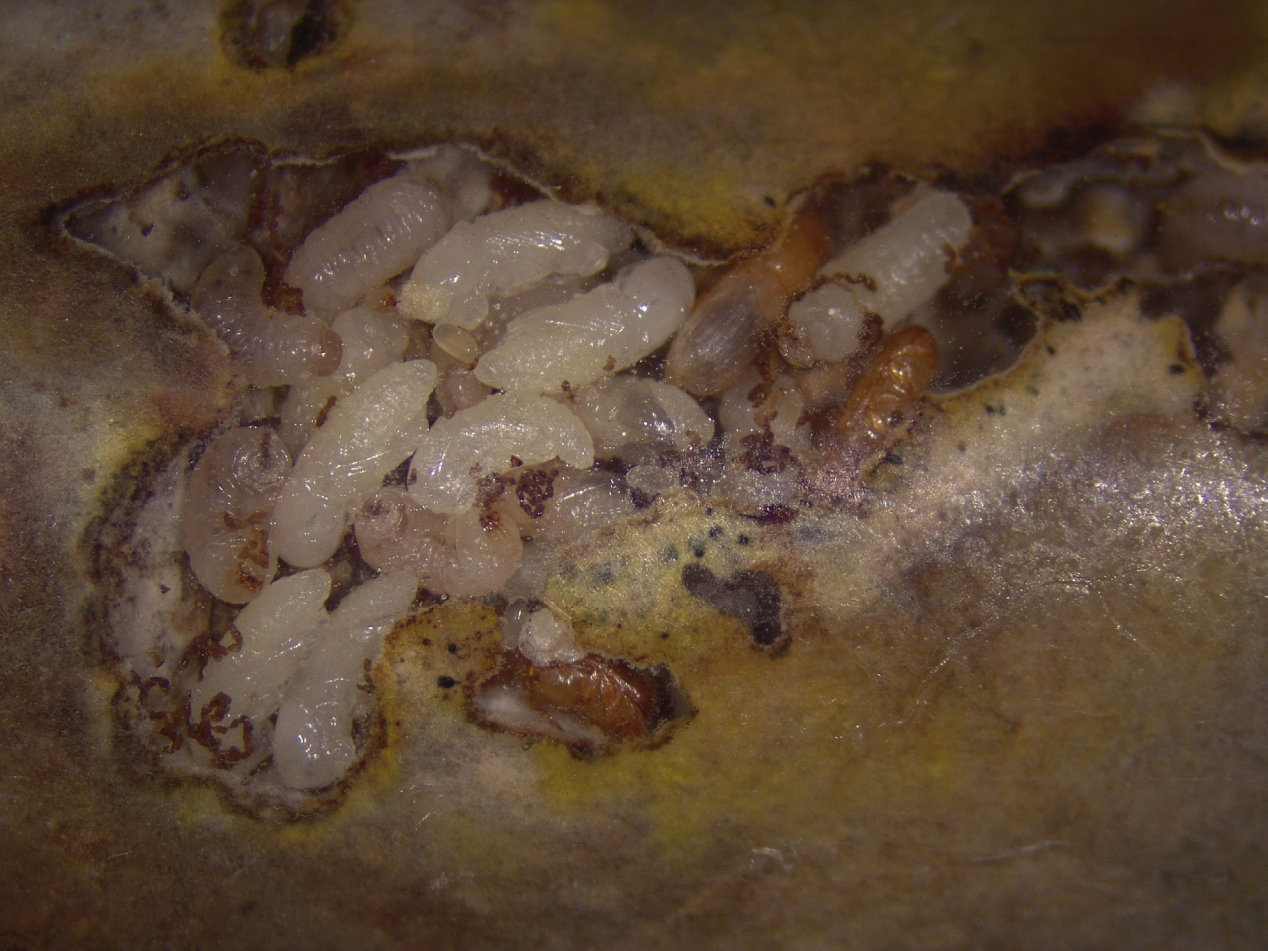
Brood chamber of a nest. Eggs, larvae, pupae and adults are visible next to each other. Because of the structure of the tunnels, some individuals are out of view in secondary tunnels.

All adopting nests originated from the SBT population, to avoid differences in the recognition threshold between populations. We also had a small number of nests from the BW population, not enough to run the mirror experiment (using BW nests as adopting ones). Each nest was used only once for this experiment to avoid changes in the recognition threshold due to previous exposure to foreign individuals. Only nests with a clearly observable brood chamber were used as adopting nests. Even in nests with a brood chamber visible, the structure of the tunnels allows some individuals to escape observation (Figure 1). We started the experiment when nests had adult females, but dispersal had not yet started (i.e., nests were 30–45 days old).

**FIGURE 2:**
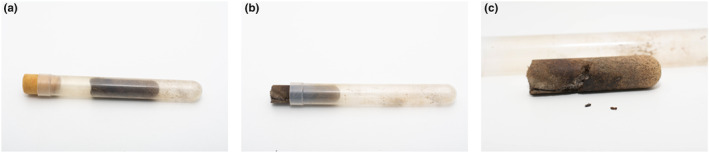
Shaking a nest out of the plastic tube. (a) Artificial medium is halfway shaken inside the tube, foam cap is still on. (b) Artificial medium is being removed from the tube. (c) Artificial medium is out of the tube, and two beetles are collected from the nest.

To permit observation, adult females were marked before introduction into a nest. The focal individual was collected from its natal nest by shaking the artificial medium out of the tube (see Figure 2) and picked up and immobilised with feather‐weight forceps. Using a fine brush, two dots of red acrylic paint (Kreul, colour n. 75205) were painted on each focal beetle, one on the extremity of the elytra and another on the pronotum. Paint was allowed to dry for a few seconds before the focal individual was introduced into an adopting nest.

The following treatments were conducted:

1. For observations of the ‘intrapopulation’ treatment, 20 adult female *X. saxesenii* from different SBT donor nests were collected, marked and introduced into 20 different SBT adopting nests. Marked females were introduced by shaking the nests out of the tubes and placing the females in the brood chambers. Afterwards, nests were pushed back into the tubes (Figure 2). Subsequently, the tube was re‐plugged and used for behavioural observations (Table 1).
2. For observations of the ‘interpopulation’ treatment, 22 adult female *X. saxesenii* were collected from the BW population, marked and introduced into adopting SBT nests.
3. In the ‘interspecific’ treatment, 20 *X. germanus* females were introduced into SBT *X. saxesenii* nests.
4. In the control group, 20 *X. saxesenii* females were collected from the adopting nest after shaking the nest out of the tube, painted and put back into their original nests.

**TABLE 1:** Ethogram of the observed behaviours of larvae (L) and adult females (F). Modified from Biedermann and Taborsky (2011).

Behaviour	Shown by	Description
Blocking	F	Blocking of the entrance tunnel using the whole body
Resting	L, F	Staying inactive in a tunnel or the brood chamber
Feeding	L, F	Grazing on the fruiting bodies, or the fungal layer covering nest walls with the maxillae and/or the mandibles
Cleaning	L, F	Grazing on the wall of the plastic tube with the maxillae and/or the mandibles
Digging	L, F	Enlarging the brood chamber or tunnels by chewing into the fungus‐infested substrate
Shuffling	F	Moving sawdust and faeces with the body (larvae) or the hind legs (adults)
Balling	L	Forming balls of sawdust and faeces by repeated ventral body contractions. Only larvae display this behaviour
Walking	L, F	Locomotion within the nest
Cannibalising	L, F	Cannibalising nestmates (usually killing and feeding on larvae)
Grooming	L, F	Cleaning of nestmates using mouthparts
Copulating	F	Mating with nestmate

A second round of introductions was performed using the same method. During the second round of introduction, the presence of the introduced female and its condition were recorded after 24 h. In both rounds, some marked females were not found after 24 h, most likely because they lost their paint markings. As we marked the introduced individuals on hard body parts (elytras and pronotum), it is not possible that an introduced female was killed and completely cannibalised within 24 h without leaving any trace. We also never found a marked female that had been partially cannibalised. However, we did not have absolute certainty about the marked females that were not found after 24 h, so these individuals were excluded from the analysis, resulting in 26, 25, 24 and 28 observations for the control, intrapopulation, interpopulation and interspecific treatments, respectively.

### 2.5 Behavioural observations

Two kinds of behavioural observations were conducted, during which we distinguished between 11 different types of behaviours, modified from Biedermann and Taborsky (2011) (see Table 1). The observer was not blind to the experimental treatment due to handling. Focal animal observations focused on the behavioural responses of introduced females. During such a focal observation, one person observed and recorded the behaviour of a marked female for 30 min immediately after its introduction into an adopting nest. Aggressiveness against non‐nestmates typically happens seconds or minutes after detection of intruders (Cappa et al., 2014, 2016).

Scan observations focused on the behavioural responses of individuals facing the introduction of a foreigner. During a scan observation, one person browsed the entire nest and recorded the number of eggs, larvae, pupae and adults. The observer also recorded the behaviours of all larvae and adult females. Scan observations were conducted as quickly as possible, usually taking two to three minutes, to get a snapshot of the activity within the nests. We did not discriminate between the three larval stages. Adult beetles were sexed on the basis of morphology and size. Scan observations were done 2 and 24 h after the introduction of the foreign female. In other social insects, aggressiveness against non‐nestmates is known to happen rapidly (Beye et al., 1998; Blight et al., 2012; Cappa et al., 2014; Jutsum et al., 1979; Lahav et al., 1999). Immediately after the last observation (i.e., 24 h), nests were opened, and the presence of the introduced female in or out of the adopting nest and its condition (alive or dead) were recorded. At the time of the experiment, no female dispersed from its native nest, so we interpreted the dispersal of introduced females as the result of fleeing the adopting nest or being evicted from it.

### 2.6 Statistical analysis

All statistical analyses were performed with R version 4.0.2 (R Core Team, 2022) using the RStudio interface version 1.3.1073 and the R packages “ggplot2” (Wickham, 2016), “randomForest” (Liaw & Wiener, 2002), “vegan” (Oksanen et al., 2019), “MASS” (Ripley et al., 2002) and “car” (Fox et al., 2019). To analyse the relative importance of individual compounds in population profiles, a random forest model was applied. We used the relative abundances of peaks (the proportion that each peak represents relative to the sum of all peaks) as the input data and the population as the grouping variable. The model built 500 trees to determine the importance of each peak in discriminating between the two groups. The number of input variables randomly selected to build each split of the tree (denoted by *mtry*) was four. To analyse the chemical distance between population profiles, we used the relative abundance of the peaks as a response variable for a permutational analysis of variance (PERMANOVA) based on Euclidean distances. To analyse differences in *n*‐alkanes, monomethyl alkanes, dimethyl alkanes and trimethyl alkanes in population profiles, a series of GLMs were applied, using the proportion of a given substance class as the response and the populations as the predictor. We also calculated the weighted average chain length of *n‐*alkanes in the two populations. It describes the average chain length of the alkanes composing the profile, each alkane weighted by its relative abundance. To analyse differences in individual hydrocarbons, a series of GLMs were applied, using the proportion of a given substance as the response and the populations as the predictor.

To investigate how introduced individuals from different origins behave in a foreign nest, we analysed each behaviour separately. We used a series of Wilcoxon rank‐sum tests with Bonferroni correction to compare the intrapopulation and interpopulation treatment groups to the control, with each test using one of the behaviours as the response variable. The total time of each respective behaviour recorded during focal observations was used for these tests.

To analyse the dispersal rate of introduced individuals, we used a series of Fisher's exact tests, comparing each treatment to the control. To analyse the survival of introduced individuals, we used a series of Fisher's exact tests, comparing each treatment to the control and comparing the pooled data from the three treatments with *X. saxesenii* (control, ‘intrapopulation’ and ‘interpopulation’) to the ‘interspecific’ treatment.

To analyse the behaviour of entire nests after the introduction of a foreign individual, four PERMANOVAs were used, with Holm correction for multiple testing. The first and second PERMANOVAs used larval behaviours observed at H + 2 and H + 24 as the response and the treatment groups as the predictor, comparing each treatment group to the control. The third and fourth PERMANOVAs used adult behaviours observed at H + 2 and H + 24 as the response. PERMANOVAS are multivariate tests, allowing comparisons of the whole behavioural pattern. A series of GLMs was conducted to investigate the effects of each larval and adult behaviour separately. In each of the GLMs, we used one of the behaviours as the response and the treatment groups as the predictor, comparing each treatment group against the control. The behaviours recorded during scan observations were transformed into binary data.

## 3 RESULTS

### 3.1 CHC profiles

**FIGURE 3:**
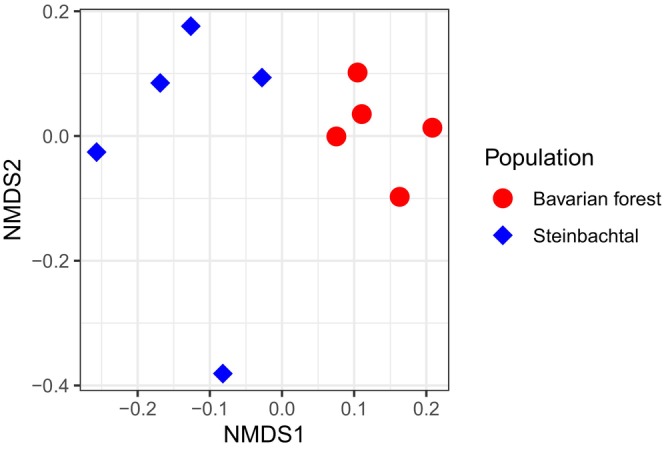
Nonmetric multidimensional scaling analysis of the CHC profiles of *X. saxesenii* adult females from the two populations. The two populations are statistically distinct (PERMANOVA, *F* = 6.607, *p* = .009; *N* = 5 × 20 adult females/population).

**FIGURE 4:**
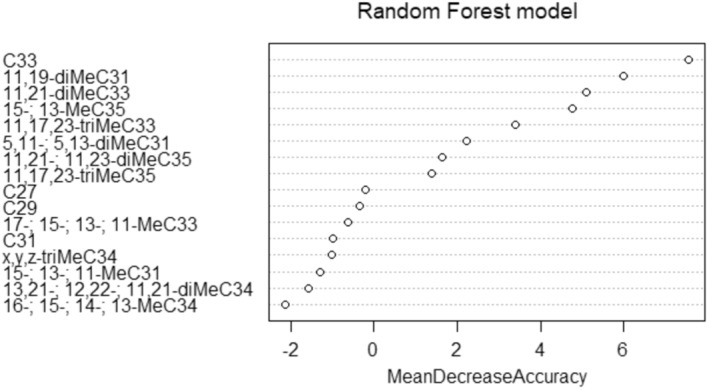
Relative importance of individual cuticular hydrocarbons for the differentiation of the two populations. Cuticular hydrocarbons playing a large part in inter‐population differentiation are ordered from top to bottom.

Sixteen compounds were characterised in the CHC profile of *X. saxesenii* females. Dimethyl alkanes were the most common compounds (*N* = 5), followed by n‐alkanes (*N* = 4), monomethyl alkanes (*N* = 4) and trimethyl alkanes (*N* = 3) (Table 2). Females from the same population had CHC profiles that were more similar to each other than to those from different populations (PERMANOVA, *F* = 6.607, *p* = .009) (Figure 3). The CHC composition represents a clear species‐specific profile with only quantitative differences in its composition between populations. The compounds explaining most of the differences in CHC profiles across populations, revealed by the random forest model, are shown in Figure 4. With the exception of tritriacontane (C33; *n*‐alkane), the compounds with the greatest contribution to the separation of the populations by CHC are methyl‐branched hydrocarbons.

**TABLE 2:** CHCs present in the profiles of adult females of *X. saxesenii* caught in the Steinbachtal forest (SBT) and Bavarian forest national park (BW).

Compound	Average retention time (min)	Mean ± SE, STB population	Mean ± SE, BW population	*t*‐Value	*p*‐Value
C27	37.8352	0.45 ± 0.12	0.25 ± 0.04	1.527	.165
C29	40.5781	1.05 ± 0.23	0.68 ± 0.11	1.466	.181
C31	43.1603	0.35 ± 0.08	0.23 ± 0.04	1.471	.180
15‐; 13‐; 11‐MeC31	43.5341	1.29 ± 0.34	1.79 ± 0.15	−1.347	.215
11,19‐diMeC31	43.9033	3.01 ± 0.23	5.64 ± 0.19	−8.917	<.001
5,11‐; 5,13‐diMeC31	44.7374	0.4 ± 0.08	0.6 ± 0.03	−2.352	.047
C33	45.1109	2.08 ± 0.1	3.16 ± 0.1	−7.937	<.001
17‐; 15‐; 13‐; 11‐MeC33	45.9272	10.55 ± 1.34	11.79 ± 0.55	−0.857	.416
11,21‐diMeC33	46.2998	41.14 ± 0.77	44.92 ± 1.23	−2.607	.031
11,17,23‐triMeC33	46.5663	17.12 ± 1.37	10.51 ± 1.9	2.825	.022
16‐; 15‐; 14‐; 13‐MeC34	47.0677	1.35 ± 0.16	1.46 ± 0.12	−0.532	.609
13,21‐; 12,22‐; 11,21‐diMeC34	47.4145	2.76 ± 0.48	3.43 ± 0.47	−0.984	.354
x,y,z‐triMeC34	47.6807	1.24 ± 0.21	1.04 ± 0.13	0.837	.427
15‐; 13‐MeC35	48.1828	2.44 ± 0.14	1.71 ± 0.12	3.887	.005
11,21‐; 11,23‐diMeC35	48.5408	10.07 ± 0.66	8.2 ± 0.51	2.233	.056
11,17,23‐triMeC35	48.8129	2.64 ± 0.3	3.08 ± 0.21	−1.207	.262

*Note*: For each compound, its average contribution to the total profile is expressed as a percentage, with a standard error. The *t*‐values and *p*‐values from the GLMs are also reported.

Populations did not differ in (i) *N‐*alkanes (GLM, *t* = −0.868, *p* = .411), which made up 4% of the CHC profile of the SBT population and 4.38% of the profile of the BW population (ii). Monomethyl alkanes (*t* = −0.594, *p* = .569) made up 15.97% and 17.01% of the SBT and BW population profiles, respectively. Dimethyl alkanes were significantly more abundant in the SBT population (58.59% vs. 63.77% in the SBT vs. BW population; *t* = −2.816, *p* = .023), while trimethyl alkanes were more abundant in the BW population (21.44% vs. 14.84% in the SBT vs. BW population; *t* = 2.713, *p* = .027). The weighted average chain length of *n‐*alkanes was 31.17 in the SBT population and 31.92 in the BW population.

Populations differed in C33 (GLM, *t* = −7.937, *p* < .001), which made up 2.08% of the CHC profile of the SBT population and 3.16% of the profile of the BW population; 11,19‐diMeC31 (*t* = −8.917, *p* < .001) made up 3.01% and 5.64% of the SBT and BW population profiles, respectively; 11,21‐diMeC33 (*t* = −2.607, *p* = .031) made up 41.14% and 44.92% of the population profiles; 15‐; 13‐MeC35 (*t* = 3.887, *p* = .005) made up 2.44% and 1.71% of the profiles; 11,17,23‐triMeC33 (*t* = 2.825, *p* = .022) made up 17.12% and 10.51% of the SBT and BW population profiles; and 5,11‐; 5,13‐diMeC31 (*t* = −2.352, *p* = .047) made up 0.4% and 0.6% of the population profiles. Other hydrocarbons did not differ between populations (all *p*‐values >.05) (Table 2).

### 3.2 Behaviour of the introduced females

**FIGURE 5:**
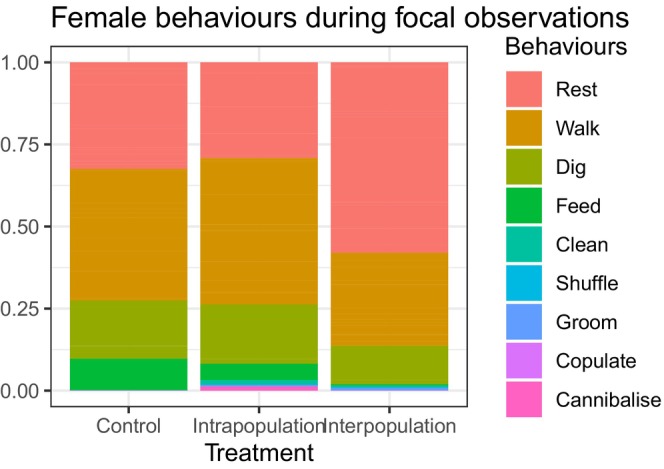
Behaviours of the introduced adult females during the focal observations. Each behaviour is expressed as a proportion of the total duration of the observation.

In control nests, resting, walking, digging and feeding were the most common behaviours of the introduced females. Introduced females spent 32.46% of their time resting, 40.13% walking, 17.70% digging and 9.54% feeding (*N* = 20) (Table 3, Figure 5).

**TABLE 3:** Behaviours of the introduced individuals during the 30‐min focal observations (control treatment *N* = 20, intrapopulation treatment *N* = 20, interpopulation treatment *N* = 22).

Behaviour	Treatment	Frequency
Walk	Control	32.46
	Intrapopulation	29.15
	Interpopulation	58.01
	Interspecies	55.85
Walk	Control	40.13
	Intrapopulation	44.60
	Interpopulation	28.37
	Interspecies	36.51
Dig	Control	17.70
	Intrapopulation	18.05
	Interpopulation	11.74
	Interspecies	3.74
Feed	Control	9.54
	Intrapopulation	5.03
	Interpopulation	0.32
	Interspecies	2.30
Clean	Control	0.00
	Intrapopulation	1.43
	Interpopulation	0.60
	Interspecies	1.54
Shuffle	Control	0.00
	Intrapopulation	0.00
	Interpopulation	0.12
	Interspecies	0.00
Groom	Control	0.17
	Intrapopulation	0.53
	Interpopulation	0.85
	Interspecies	0.05
Cannibalise	Control	0.00
	Intrapopulation	1.22
	Interpopulation	0.00
	Interspecies	0.00

*Note*: The average frequencies of the behaviours in the different treatment groups are expressed as a percentage of the total duration of the observation. The time when the focal individual was out of view was not included in the observation.

Introduced females in the intrapopulation treatment exhibited similar behaviours to the females in the control group; there was no significant effect of treatment (Wilcoxon rank‐sum tests, all *p*‐values >.897) (Table 4). Introduced females in the interpopulation treatment also exhibited similar behaviours to the females in the control group (all *p*‐values >.171) (Table 4). The introduced *X. germanus* behaviours in the interspecific treatment were not compared to *X. saxesenii* behaviours. It is difficult and potentially confusing to compare the behaviours of different species. A recent study describing the behaviours of *X. germanus* in their nests shows that this species has a behavioural repertoire distinct from the one of *X. saxesenii* (Milbrath et al., 2024). During the focal observations, no beetle dispersed from the nest.

**TABLE 4:** Differences in behaviours of the introduced individuals during the 30‐min focal observations (control treatment *N* = 20, intrapopulation treatment *N* = 20, interpopulation treatment *N* = 22).

Behaviour	Parameter	W‐value	*p*‐Value
Rest	Control—Intrapopulation	206.5	1
	Control—Interpopulation	174.5	.768
Walk	Control—Intrapopulation	188	1
	Control—Interpopulation	237	1
Dig	Control—Intrapopulation	198	1
	Control—Interpopulation	261	.603
Feed	Control—Intrapopulation	203.5	1
	Control—Interpopulation	266.5	.171
Clean	Control—Intrapopulation	190	1
	Control—Interpopulation	210	1
Shuffle	Control—Intrapopulation	200	NA
	Control—Interpopulation	210	NA
Groom	Control—Intrapopulation	179.5	.897
	Control—Interpopulation	210	1
Cannibalise	Control—Intrapopulation	190	NA
	Control—Interpopulation	220	NA

*Note*: Wilcoxon rank‐sum tests with Bonferroni correction were used to compare the intrapopulation and interpopulation treatment groups to the control.

### 3.3 Dispersal and survival of introduced females

**FIGURE 6:**
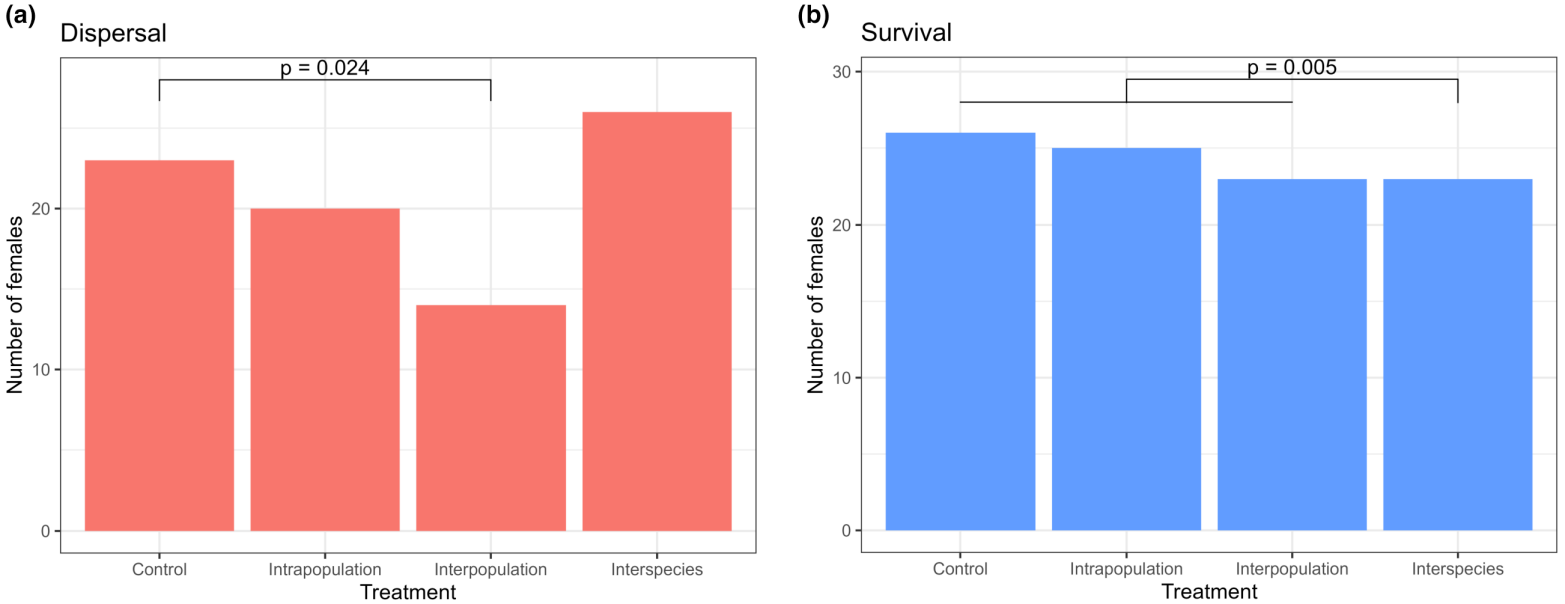
Dispersal (a) and survival (b) of introduced *X. saxesenii* adult females 24 h after experimental introduction within the natal nest (control), a nest of the same population (intrapopulation) and a foreign population (interpopulation). In the “interspecies” treatment, an *X. germanus* adult female was introduced into a *X. saxesenii* nest. The number of introduced individuals that were observed within the nest (a) or were observed alive (b) was compared with Fisher exact tests.

After females were introduced into nests, they either remained within the nest or dispersed in the next 24 h. 12% of females in the control treatment dispersed (3 out of 26), 20% (5 out of 25) in the ‘intrapopulation’ treatment, 42% (10 out of 24) in the ‘interpopulation’ treatment and 7% (2 out of 28) in the ‘interspecific’ treatment (Figure 6). The dispersal of females did not differ from the control in the ‘intrapopulation’ treatment (Fisher's exact test, *p* = .465; *N* = 26 vs. 25) and ‘interspecific’ treatment (*p* = .663; *N* = 26 vs. 28), but was higher in the ‘interpopulation’ treatment (*p* = .024; *N* = 26 vs. 24).

All females of *X. saxesenii* survived, except one that was likely killed during the nest manipulation. However, five out of 30 observed *X. germanus* females in the ‘interspecific’ treatment died. The survival of females did not differ from the control in the ‘intrapopulation’ treatment (Fisher's exact test, *p* = 1; *N* = 26 vs. 25) and ‘interpopulation’ treatment (*p* = .48; *N* = 26 vs. 24). We see a trend towards a higher death rate in the ‘interspecific’ treatment (*p* = .052; *N* = 26 vs. 28). If all *X*. *saxesenii* treatments were combined, there was a significantly higher likelihood of death in the ‘interspecific’ treatment (Fisher's exact test: *p* = .005; *N* = 75 vs. 28). However, no aggressive behaviour directed towards the introduced individuals was observed, and the dead individuals showed no visible injuries.

### 3.4 Behaviour of receiving individuals in the adopting nests

Firstly, we analysed the whole behavioural pattern of the receiving nests, considering all behaviours in a multi‐variate analysis. Secondly, each larval and adult behaviour was analysed separately.

**FIGURE 7:**
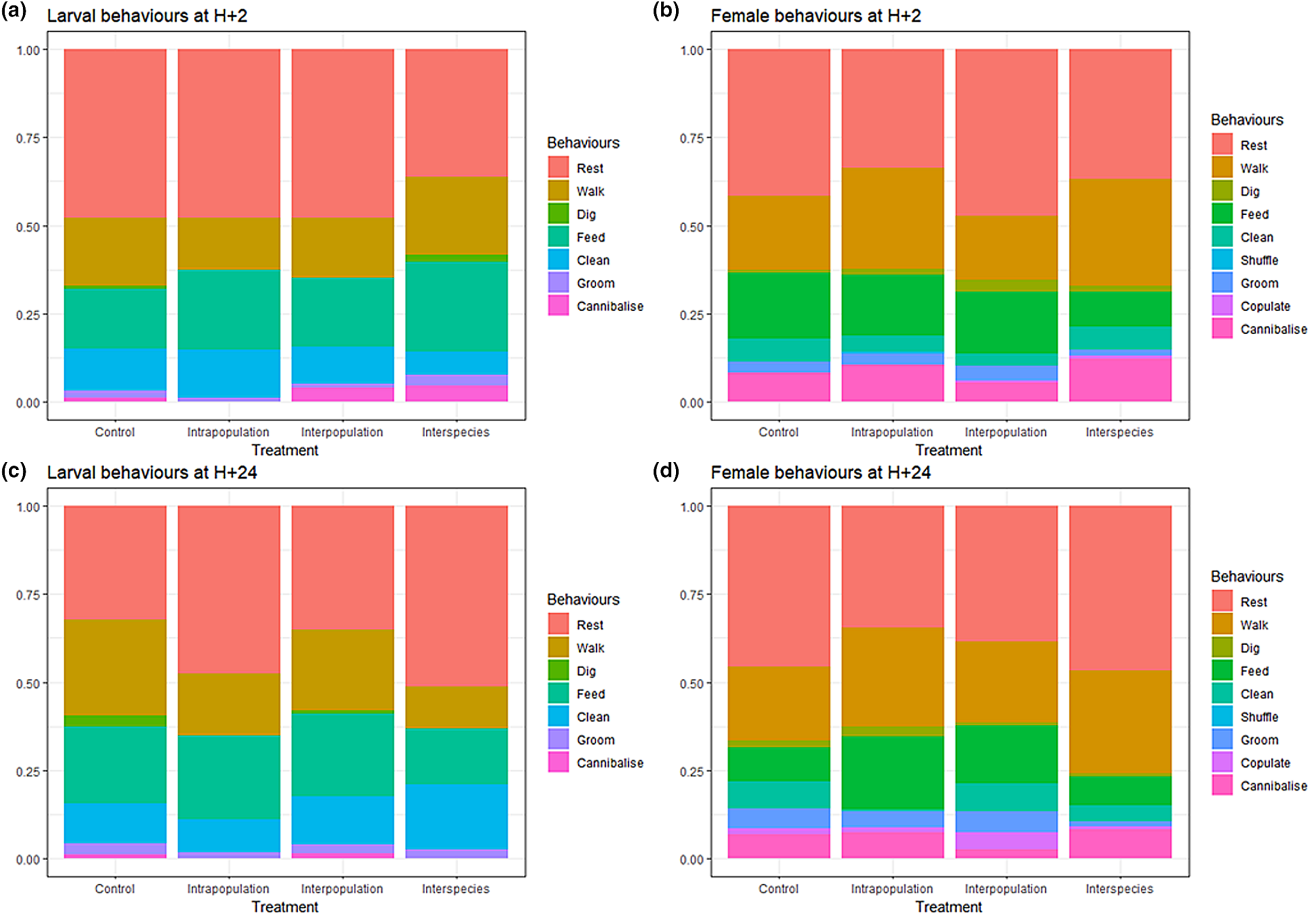
Behaviour of the adopting larvae (a, c) and adult females (b, d) during the scan observations two (a, b) or 24 h after the manipulation (c, d). Individual behaviours are shown as a proportion of the observed behaviours.

The behavioural pattern of receiving females in the ‘intrapopulation’ treatment did not differ from the behavioural pattern in the control group after 2 h (PERMANOVA, *F* = 1.789, *p* = .18) and after 24 h (*F* = 1.316, *p* = .249) (Table 5, Figure 7). No behaviour changed in frequency after 2 h (Table 6), but after 24 h, the receiving females in the intrapopulation treatment fed more than the females in the control group (20.59% of observed behaviours in the intrapopulation treatment, 9.52% in the control; GLM, *z* = 2.286, *p* = .023) and cleaned less (0.74% vs. 7.62%; *z* = −2.255, *p* = .024) (Table 6). Receiving larvae did not change their behavioural pattern after 2 h (PERMANOVA, *F* = 0.205, *p* = .732) and after 24 h (*F* = 1.407, *p* = .26) (Figure 7; Table 7).

**TABLE 5:** Behaviours of the receiving receiver adult females in adopting nests during scan observations at H + 2 and H + 24 (control treatment *N* = 20, intrapopulation treatment *N* = 20, interpopulation treatment *N* = 22, interspecies treatment *N* = 20).

Behaviour	Treatment	Frequency	
		H + 2	H + 24
Rest	Control	41.67	45.71
	Intrapopulation	33.83	34.56
	Interpopulation	47.30	38.41
	Interspecies	36.89	46.62
Walk	Control	20.83	20.95
	Intrapopulation	28.57	27.94
	Interpopulation	18.24	23.18
	Interspecies	30.33	29.32
Dig	Control	1.04	1.90
	Intrapopulation	1.50	2.94
	Interpopulation	3.38	0.66
	Interspecies	1.64	0.75
Feed	Control	18.75	9.52
	Intrapopulation	17.29	20.59
	Interpopulation	17.57	16.56
	Interspecies	9.84	8.27
Clean	Control	6.25	7.62
	Intrapopulation	4.51	0.74
	Interpopulation	3.38	7.95
	Interspecies	6.56	4.51
Shuffle	Control	0.00	0.00
	Intrapopulation	0.75	0.00
	Interpopulation	0.00	0.00
	Interspecies	0.00	0.00
Groom	Control	3.13	5.71
	Intrapopulation	3.01	4.41
	Interpopulation	4.05	5.96
	Interspecies	1.64	1.50
Copulate	Control	0.00	1.90
	Intrapopulation	0.00	1.47
	Interpopulation	0.68	4.64
	Interspecies	0.82	0.75
Cannibalise	Control	8.33	6.67
	Intrapopulation	10.53	7.35
	Interpopulation	5.41	2.65
	Interspecies	12.30	8.27

*Note*: The average frequencies of the behaviours in the different treatment groups are expressed as a percentage of the total number of behaviours observed.

**TABLE 6:** Differences in behaviour of the receiver adult females in adopting nests during scan observations at H + 2 and H + 24 (control treatment *N* = 20, intrapopulation treatment *N* = 20, interpopulation treatment *N* = 22, interspecies treatment *N* = 20).

Behaviour	Parameter	H + 2			H + 24		
		Coeff ± SE	*z*	*p*	Coeff ± SE	*z*	*p*
Rest	Mean	−0.337 ± 0.207	−1.625		−0.172 ± 0.196	−0.877	
	Intrapopulation	−0.334 ± 0.277	−1.209	.227	−0.466 ± 0.266	−1.753	.38
	Interpopulation	0.228 ± 0.265	0.863	.388	−0.3 ± 0.258	−1.166	.08
	Interspecies	−0.201 ± 0.279	−0.718	.473	−0.036 ± 0.262	0.139	.89
Walk	Mean	−1.335 ± 0.251	−5.312		−1.328 ± 0.24	−5.537	
	Intrapopulation	0.419 ± 0.316	1.324	.185	0.38 ± 0.307	1.241	.215
	Interpopulation	−0.165 ± 0.329	−0.501	.616	0.13 ± 0.308	0.421	.374
	Interspecies	0.503 ± 0.319	1.576	.115	0.448 ± 0.306	1.463	.143
Dig	Mean	−4.554 ± 1.005	−4.53		−3.942 ± 0.714	−5.521	
	Intrapopulation	0.372 ± 1.232	0.302	.763	0.445 ± 0.876	0.508	.611
	Interpopulation	1.201 ± 1.103	1.088	.277	−1.069 ± 1.231	−0.868	.385
	Interspecies	0.460 ± 1.232	0.373	.709	−0.941 ± 1.232	−0.764	.445
Feed	Mean	−1.466 ± 0.261	−5.608		−2.251 ± 0.332	−6.772	
	Intrapopulation	−0.099 ± 0.348	−0.284	.777	0.901 ± 0.394	2.286	.022
	Interpopulation	−0.08 ± 0.339	−0.235	.815	0.634 ± 0.398	1.592	.111
	Interspecies	−0.749 ± 0.401	−1.868	.062	−0.155 ± 0.458	−0.338	.735
Clean	Mean	−2.709 ± 0.422	−6.423		−2.495 ± 0.368	−6.783	
	Intrapopulation	−0.344 ± 0.594	−0.58	.562	−2.41 ± 1.069	−2.255	.024
	Interpopulation	−0.645 ± 0.62	−1.04	.298	0.046 ± 0.475	0.096	.923
	Interspecies	0.051 ± 0.558	0.092	.927	−0.557 ± 0.557	−1.001	.317
Shuffle	Mean	−2.464 e+01 ± 1.391 e+03	−0.002		−27.684 ± 60768.059	0	
	Intrapopulation	1.976 e+01 ± 1.391 e+03	0.001	.999	−0.102 ± 82756.31	0	1
	Interpopulation	−1.008 e‐01 ± 1.823 e+04	0	1	−0.128 ± 81301.773	0	1
	Interspecies	−1.693 e‐02 ± 1.866 e+04	0	1	−0.063 ± 82447.877	0	1
Groom	Mean	−3.434 ± 0.587	−5.854		−2.803 ± 0.42	−6.668	
	Intrapopulation	−0.04 ± 0.776	−0.051	.959	−0.272 ± 0.593	−0.46	.646
	Interpopulation	0.27 ± 0.72	0.375	.708	−0.045 ± 0.543	0.082	.934
	Interspecies	−0.66 ± 0.923	−0.715	.474	−1.379 ± 0.827	−1.667	.096
Copulate	Mean	−23.645 ± 8436.011	−0.003		−3.942 ± 0.714	−5.521	
	Intrapopulation	−0.143 ± 11420.852	0	1	−0.263 ± 1.009	−0.261	.794
	Interpopulation	18.654 ± 8436,011	0.002	.998	0.918 ± 0.812	1.13	.258
	Interspecies	18.849 ± 8436.011	0.002	.998	−0.941 ± 1.232	−0.764	.445
Cannibalise	Mean	−2.398 ± 0.369	−6.494		−2.639 ± 0.391	−6.746	
	Intrapopulation	0.258 ± 0.465	0.555	.579	0.105 ± 0.511	0.206	.837
	Interpopulation	−0.464 ± 0.518	−0.896	.37	−0.965 ± 0.64	−1.507	.132
	Interspecies	0.433 ± 0.461	0.94	.347	0.233 ± 0.502	0.464	.643

*Note*: The behaviours recorded during scan observations were transformed into binary data. GLMs were used to compare each treatment group to the control.

**TABLE 7:** Behaviours of the receiving receiver larvae in adopting nests during scan observations at H + 2 and H + 24 (control treatment *N* = 20, intrapopulation treatment *N* = 20, interpopulation treatment *N* = 22, interspecies treatment *N* = 20).

Behaviour	Treatment	Frequency	
		H + 2	H + 24
Rest	Control	48.00	32.29
	Intrapopulation	47.73	47.62
	Interpopulation	47.78	35.14
	Interspecies	36.26	51.32
Walk	Control	19.00	27.08
	Intrapopulation	14.77	17.46
	Interpopulation	17.22	22.97
	Interspecies	21.98	11.84
Dig	Control	1.00	3.13
	Intrapopulation	0.00	0.00
	Interpopulation	0.00	0.68
	Interspecies	2.20	0.00
Feed	Control	17.00	21.88
	Intrapopulation	22.73	23.81
	Interpopulation	19.44	23.65
	Interspecies	25.27	15.79
Clean	Control	12.00	11.46
	Intrapopulation	13.64	9.52
	Interpopulation	10.56	13.51
	Interspecies	6.59	18.42
Groom	Control	2.00	3.13
	Intrapopulation	1.14	1.59
	Interpopulation	1.11	2.70
	Interspecies	3.30	2.63
Cannibalise	Control	1.00	1.04
	Intrapopulation	0.00	0.00
	Interpopulation	3.89	1.35
	Interspecies	4.40	0.00

*Note*: The average frequencies of the behaviours in the different treatment groups are expressed as a percentage of the total number of behaviours observed.

After 2 h (PERMANOVA, *F* = 2.01, *p* = .151) as well as after 24 h (*F* = 1.107, *p* = .288), the behaviour of receiving females in the ‘interpopulation’ treatment did not differ from the behavioural pattern in the control group. Receiving larvae also did not change their behavioural pattern after 2 h (PERMANOVA, *F* = 2.7, *p* = .113) and after 24 h (*F* = 0.936, *p* = .362 at H + 24) (Table 7, Figure 7). No behaviour changed significantly in frequency after the introduction (Tables 6 and 8).

**TABLE 8:** Differences in behaviour of the receiver larvae in adopting nests during scan observations at H + 2 and H + 24 (control treatment *N* = 20, intrapopulation treatment *N* = 20, interpopulation treatment *N* = 22, interspecies treatment *N* = 20).

Behaviour	Parameter	H + 2			H + 24		
		Coeff ± SE	*z*	*p*	Coeff ± SE	*z*	*p*
Rest	Mean	−0.08 ± 0.2	−0.4		−0.74 ± 0.218	−3.392	
	Intrapopulation	−0.011 ± 0.293	−0.037	.97	0.645 ± 0.334	1.934	.053
	Interpopulation	−0.009 ± 0.25	−0.036	.972	0.127 ± 0.278	0.458	.647
	Interspecies	−0.484 ± 0.296	−1.635	.102	0.793 ± 0.317	2.504	.012
Walk	Mean	−1.45 ± 0.255	−5.688		−0.99 ± 0.23	−4.312	
	Intrapopulation	−0.303 ± 0.394	−0.768	.443	−0.563 ± 0.404	−1.395	.163
	Interpopulation	−0.12 ± 0.322	−0.372	.71	−0.219 ± 0.302	−0.728	.467
	Interspecies	0.183 ± 0.359	0.51	.61	−1.017 ± 0.423	−2.405	.016
Dig	Mean	−4.595 ± 1.005	−4.572		−3.434 ± 0.587	−5.854	
	Intrapopulation	−17.951 ± 5085.473	−0.004	.997	−18.965 ± 5586.279	−0.003	.997
	Interpopulation	−18.398 ± 4447.497	−0.004	.997	−1.556 ± 1.162	−1.339	.181
	Interspecies	0.8 ± 1.233	0.648	.517	−19.252 ± 5869.622	−0.003	.997
Feed	Mean	−1.586 ± 0.266	−5.956		−1.273 ± 0.247	−5.156	
	Intrapopulation	0.362 ± 0.368	0.983	.326	0.11 ± 0.385	0.285	.776
	Interpopulation	0.164 ± 0.326	0.504	.614	0.101 ± 0.314	0.322	.748
	Interspecies	0.502 ± 0.359	1.396	.163	−0.401 ± 0.4	−1.003	.316
Clean	Mean	−1.992 ± 0.308	−6.475		−2.045 ± 0.32	−6.382	
	Intrapopulation	0.147 ± 0.437	0.335	.737	−0.207 ± 0.536	−0.386	.7
	Interpopulation	−0.145 ± 0.392	−0.369	.712	0.189 ± 0.401	0.47	.638
	Interspecies	−0.659 ± 0.523	−1.26	.208	0.557 ± 0.436	1.276	.202
Shuffle	Mean	−2.759 e+01 ± 5.954 e+04	0		−2.759 e+01 ± 6.07 e+04	0	
	Intrapopulation	4.831 e‐02 ± 8.593 e+04	0	1	1.925 e‐01 ± 9.119 e+04	0	1
	Interpopulation	−3.831 e‐01 ± 8.05 e+04	0	1	−3.728 e‐01 ± 8.458 e+04	0	1
	Interspecies	−1.171 e‐02 ± 8.902 e+04	0	1	−9.401 e‐02 ± 9.38 e+04	0	1
Groom	Mean	−3.892 ± 0.714	−5.449		−3.434 ± 0.587	−5.854	
	Intrapopulation	−0.574 ± 1.234	−0.465	.642	−0.693 ± 1.166	−0.594	.552
	Interpopulation	−0.597 ± 1.008	−0.592	.554	−0.15 ± 0.775	−0.193	.847
	Interspecies	0.513 ± 0.925	−0.555	.579	−0.177 ± 0.926	−0.191	.848
Cannibalise	Mean	−4.595 ± 1.005	−4.572		−4.554 ± 1.005	−4.53	
	Intrapopulation	−15.951 ± 1870.841	−0.009	.993	−17.845 ± 5586.279	−0.003	.997
	Interpopulation	1.388 ± 1.076	1.289	.197	0.263 ± 1.232	0.214	.831
	Interspecies	1.516 ± 1.128	1.344	.179	−18.132 ± 5869.622	−0.003	.998

*Note*: The behaviours recorded during scan observations were transformed into binary data. GLMs were used to compare each treatment group to the control.

The behavioural pattern of receiving females in the ‘interspecies’ treatment also did not differ from the behavioural pattern in the control group after 2 h (PERMANOVA, *F* = 0.844, *p* = .386) and after 24 h (*F* = 0.949, *p* = .364). No behaviour changed in frequency after two or 24 h (Table 6). The behavioural pattern of receiving larvae did not differ after 2 h (PERMANOVA, *F* = 0.168, *p* = .764) and after 24 h (*F* = 0.491, *p* = .537) (Figure 7). No behaviour changed in frequency after 2 h (Table 8), but after 24 h, the receiving larvae in the interspecific treatment rested more than the larvae in the control group (51.32% of observed behaviours in the interspecific treatment, 32.29% in the control; GLM, *z* = 2.504, *p* = .012) and walked less (11.84% vs. 27.08%; GLM, *z* = −2.405, *p* = .016) (Table 8).

## 4 DISCUSSION

The analysis of cuticular hydrocarbons revealed strong differences between two populations of *X. saxesenii*, providing possible cues for nestmate recognition between individuals of these populations. Contrary to expectations, these chemical differences did not induce aggressive behavioural responses, as we recorded none during the observation periods and no introduced *X. saxesenii* female was killed during the experiment. The only introduced females found dead after 24 h were of a different species and showed no visible injury. Some introduced *X. saxesenii* females were found outside of the adopting nest 24 h after introduction, presumably because they were expelled from the nest or left voluntarily. Leaving voluntarily would be in line with an aggressive response, but we did not observe the exact moment these females exited the nests. The proportion of introduced females that immediately dispersed from adopting nests increased with the dissimilarity across groups, a trend consistent with the occurrence of nestmate recognition. These females either are rejected more often by less similar adopters or they freely choose to leave less similar recipient nests.

Introduced females exhibited similar behaviours across treatments, with none of the recorded behaviours differing between the control group and the other experimental treatments. The behavioural analysis of receiving individuals did not reveal any aggressive responses to the introduction of a foreign female. Against our expectations, the overall behaviour of the adults and larvae in the receiving nests did not change, and the minor differences observed (e.g. in feeding, cleaning and walking) are most likely random and independent of the introduced individual. These differences were observed 24 h after the introduction, challenging a treatment‐induced response.

### 4.1 CHC profiles

The CHC profile of *X. saxesenii* is composed of *n*‐alkanes and methyl‐branched alkanes. Methyl‐branched alkanes, which explain most of the differences between the two populations, are used as cues for recognition processes in ants, whereas *n*‐alkanes are not important as cues or for signalling (Van Zweden et al., 2010). The quantitative differences between the female *X. saxesenii* CHC profiles of the two populations and the significance of methyl‐branched hydrocarbons for these population differences provide evidence for potential recognition cues between the two populations. Previous studies showed that chemical profiles signal reproductive abilities in bees, bumblebees, wasps, ants and termites (Blacher et al., 2013; Moore & Liebig, 2010; Richard & Hunt, 2013). The competitive ability of the foreigner may be a better predictor than relatedness for acceptance or rejection (Blacher et al., 2013; Matsuura & Nishida, 2001; Moore & Liebig, 2010). Since females of *X. saxesenii* mate quickly after emergence, foreign and native females have similar reproductive potential.

A primary function of the CHC layer is to limit water loss. Terrestrial arthropods change their CHC profiles in reaction to temperature and humidity (Hadley, 1977; Michelutti et al., 2018; Toolson & Hadley, 1979; Wang et al., 2022; Woodrow et al., 2000). Closely related species adapted to different climates have different CHC profiles (Menzel et al., 2017; Wang et al., 2022), and different populations within a species can also have different chemical profiles; this has been studied in social insects (Gévar et al., 2017; Peña‐Carrillo et al., 2021; Soares et al., 2021). Differences in CHC profiles may be adaptations to the slightly warmer and dryer climate in Würzburg as compared to Neuschönau (the yearly average temperature and precipitation: 9.9°C and 757 mm in Würzburg; 7.2°C and 1263 mm in Neuschönau (source: en.climate‐data.org)). However, the CHC profiles of the Würzburg population did not show an increase in *n‐*alkane proportions or a shift to a higher chain length in hydrocarbons as expected in an adaptation to increased drought due to lower humidity or higher temperatures (Buellesbach et al., 2018; Menzel et al., 2018). More likely, if differences are non‐adaptive, CHC profile differences may result from random genetic drift. Similarly to what happens in mountain wasps, where limited gene flow between populations can explain differences in CHC profiles (Bonelli et al., 2015), neutral mutations can quickly invade a small population of *X. saxesenii* and result in different chemical signatures because of their inbreeding habits. The CHC profiles of bark and ambrosia beetles have only been investigated in a few studies (Chen et al., 2017; Howard & Infante, 1996; Myrie, 2024; Page et al., 1990), and their variability between populations of a species is even less studied (Myrie, 2024). We focused our investigation on CHCs because their role in nestmate recognition is well characterised, but polar compounds may also trigger behavioural responses. Polar lipids are used as sex pheromones in a parasitic wasp (Kühbandner et al., 2012), but we found no polar compounds that could be used as recognition cues in this ambrosia beetle.

### 4.2 Behavioural responses

As the proportion of foreign females found outside the adopting nests increased, the genetic distinction between their adopting nest and their nest of origin (with the exception of allospecific females, as discussed in section *Interspecific treatment*) also increased. This is consistent with the subtle aggression exhibited by resident individuals and the non‐nestmates voluntarily leaving. It supports our hypothesis that *X. saxesenii* can identify nestmates and change behaviour accordingly, i.e., more different individuals trigger a stronger behavioural response as shown in other social insects (Beye et al., 1998; Blight et al., 2012; Frizzi et al., 2014; Vásquez & Silverman, 2008).

Resident individuals showed only minor behavioural changes after the introduction of the non‐nestmate. When a female from the same population was introduced to a nest, resident adult females exhibited more feeding behaviour and less cleaning behaviour. We do not interpret this as an adaptive behavioural response for two reasons: Firstly, many social insects respond quickly and aggressively to the presence of a non‐nestmate in the nest (Sturgis & Gordon, 2012). An increase in feeding behaviour does not seem to be an aggressive response towards non‐nestmates. Secondly, a small difference between CHC profiles (intrapopulation) should elicit a weaker behavioural response than a large distance (interpopulation) (Beye et al., 1998; Blight et al., 2012; Drescher et al., 2010; Frizzi et al., 2014; Holzer et al., 2006).

The absence of an aggressive behavioural response to the presence of a non‐nestmate does not mean that *X. saxesenii* is unable to differentiate between nestmates and non‐nestmates. The aggressive response to a non‐nestmate is well studied in social insects, but very few studies have investigated other behavioural responses. Interestingly, a study on Argentine ants found that non‐nestmates, which did not interact aggressively, engaged in more allogrooming than nestmates (Björkman‐Chiswell et al., 2008). In *X. saxesenii*, a behavioural response could be the observed reduction of allogrooming towards introduced females. Importantly, as antagonistic encounters are typically short or possibly subtle, we cannot exclude that the temporal resolution of our method (i.e., nests observed for the first 30 min, at 2 and 24 h) was not sufficient to detect them. Longer focal observations and continuous video capture would increase the temporal resolution of the data and the probability of observing short and subtle encounters.

In natural conditions, a narrow tunnel allows efficient blocking by the native beetles to avoid larval escapes, to regulate atmospheric nest conditions (Nuotclà et al., 2014) and to protect the nest from any intruder that is bigger than *X. saxesenii*. The protection from intruders' aspect was tested in a pilot study with a design similar to that of this study: foreign females from the same nest, from the same population, a different population and a different species were introduced and individually observed in foreign nests using a dot of red paint. Approximately 20 foreign females were tested for each treatment. Not a single one of these females was found entering the nest, even in the control group (pers. obs.). This supports the assumption that blocking the entrance tunnel is highly effective in ambrosia beetles and that there is no need to evolve a nestmate‐recognition system. The introduction of foreign individuals in the brood chamber thus bypasses the blocking female and may reflect a rather uncommon situation that only occurs if nests merge, which happens only rarely in *X. saxesenii* (Wichmann, 1954).

### 4.3 Interspecific treatment

It is noteworthy that the only individuals found dead after 24 h were *X. germanus*. Because the time between introduction and final observation was relatively short, it is unlikely that they died from starvation (they probably cannot feed on *X. saxesenii*'s fungal garden). They were not obviously wounded (no missing legs or antennae, no holes in the cuticle), so their death is most likely not the result of aggression from native individuals. A more likely cause of death is the high stress faced by these individuals. The larger size of *X. germanus* compared to *X. saxesenii* possibly resulted in individuals who might have had difficulties moving in the adopted nests. Some of the introduced *X. germanus* were observed in the same place over consecutive observations. The results would thus be biased towards non‐dispersal. Again, continuous video capture would help to understand what happened to these beetles.

### 4.4 Perspectives

A primary aim of our study was to identify and describe nestmate recognition and aggressive behaviours against non‐nestmates in *X. saxesenii*, but such behaviour was not observed. However, we cannot rule out that we missed informative behaviour. Ambrosia beetles are tiny, and even under the microscope, the exact movements of mouthparts, legs and antennae are difficult to observe. Other behaviours are subtle and not easily interpreted. For example, we cannot differentiate feeding from fungus‐tending behaviour. Also, allogrooming behaviour may comprise unseen aggressive usage of mandibles. Additionally, aggressive behaviour may have occurred before or after our observations. We did not record the behaviours continuously, and rejection may happen several hours after introduction. Social insects often react immediately to the introduction of a non‐nestmate (Sturgis & Gordon, 2012), but it appears that *X. saxesenii* does not react in the first 30 min. Future experiments should include continuous video recording to capture any behavioural response happening after the first 30 min of the foreign female introduction.

This study was based on previous personal experience by the first author with ants, wasps and mole rats, all of whom showed very obvious and strong aggressive behaviour towards non‐nestmates. Aggressiveness towards non‐nestmates was never before studied in ambrosia beetles, so we addressed this topic using the dominant theoretical framework. The lack of any obvious aggression towards non‐nestmates was quite surprising for us. Even if there is extensive empirical evidence for nestmate recognition triggering an aggressive response, other reactions are understudied (van Wilgenburg & Elgar, 2013). It is possible that females in *X. saxesenii* react to foreign individuals in very subtle ways or without immediate aggression, for example by displaying other changes in behaviour. More studies with a higher temporal resolution and specific observations of the individual blocking the entrance tunnel are needed to check whether there is really no aggression against non‐nestmates. Replicating the study with different ambrosia beetle species would be useful to test if species with a different social organisation show obvious aggressiveness towards non‐nestmates. This may be particularly interesting to test in *Ips typographus*, which has kin recognition abilities as has been proven by its preferential mating with non‐relatives (Dacquin et al., 2023).

There are also good reasons that may explain the absence of aggression in *X. saxesenii*. First, protection of the nest against non‐nestmates at the entrance tunnel through “blocking” behaviour may be very effective (Nuotclà et al., 2014), thus requiring no nestmate recognition within the nest. Another important aspect is the fitness cost imposed on the receiving colony by an intruder. If the cost is negligible, then there is no selective pressure to prevent intrusion. Since nestmate recognition is a complex system of template production, template recognition and behavioural response (Newey et al., 2010), it would readily disappear in the absence of selective pressure. However, when I started this experiment, I wanted to investigate the presence/absence of a behavioural response, not yet investigating the proximal mechanisms of such a response.

Finally, non‐nestmates may even be beneficial in inbreeding species like *X. saxesenii*, because non‐kin mating may prove beneficial. This may also be the case for *Xylosandrus germanus*, another closely related inbreeding ambrosia beetle species that suffers from outbreeding depression (Peer & Taborsky, 2005).

In conclusion, this was the first investigation of cuticular hydrocarbon differences and nestmate recognition in an ambrosia beetle species. We found clear evidence that CHC profiles differ between populations, providing potential cues for nestmate recognition. We found an increase in the dispersal of introduced females in the interpopulation treatment and subtle behavioural changes in receiver individuals. In contrast to other social insects, these behavioural reactions are not aggressive, and it is unclear if they are a response to our treatments. Further investigations are needed to understand if ambrosia beetles use CHCs for recognition and to decipher how to interpret the changes in behavioural reactions towards non‐nestmates. This would broaden our general understanding of behavioural responses towards non‐nestmates in social insect societies.

## AUTHOR CONTRIBUTIONS

**Antoine Melet:** Conceptualization (equal); formal analysis (lead); investigation (lead); writing – original draft (lead). **Viesturs Leibold:** Investigation (supporting). **Thomas Schmitt:** Writing – review and editing (equal). **Peter H. W. Biedermann:** Conceptualization (equal); writing – review and editing (lead).

## FUNDING INFORMATION

This work was funded by the German Research Foundation (DFG) (Emmy Noether grant number BI 1956/1‐1 to P.H.W.B.).

## CONFLICT OF INTEREST STATEMENT

The authors declare that the research was conducted in the absence of any commercial or financial relationship that could be a potential conflict of interest.

## Data Availability

The data and scripts that support the findings of this study are openly available in our GitHub Repository at https://github.com/AntMelet/Social‐closure‐in‐Xyleborinus‐saxesenii.
