# Correction to “Highly Diverse Cuticular Hydrocarbon Profiles but No Evidence for Aggression Towards Non‐Kin in the Ambrosia Beetle *Xyleborinus saxesenii* ”

**DOI:** 10.1002/ece3.71077

**Published:** 2025-04-03

Melet, A., V. Leibold, T. Schmitt, and P. H. W. Biedermann. 2024. “Highly Diverse Cuticular Hydrocarbon Profiles but No Evidence for Aggression Towards Non‐Kin in the Ambrosia Beetle *Xyleborinus saxesenii*.” *Ecology and Evolution* 14: e11274.

In the Correspondence section, the email of Antoine Melet is incorrect. This should be ant.melet@gmail.com.

Also, Peter Biedermann should be included as a co‐corresponding author; his email address is peter.biedermann@forento.uni-freiburg.de.

We apologize for this error.

The authors.

